# Supplementary material for: Mitochondrial ADP/ATP Carrier 1 Is Important for the Growth of Toxoplasma Tachyzoites
Source: Microbiol Spectr. 2023 May 8;11(3):e00040-23. doi: 10.1128/spectrum.00040-23 (PMC10269819; doi:10.1128/spectrum.00040-23)
Supplement: Supplemental file 1 — Supplemental material. Download spectrum.00040-23-s0001.pdf, PDF file, 5.9 MB [file spectrum.00040-23-s0001.pdf]

Mitochondrial ADP/ATP carrier 1 is essential for the growth of *Toxoplasma* tachyzoites

Jiahui Qian [1], Tongjie Zhao [1], Liyu Guo [1], Senyang Li [2], Zhengming He [1], Mingfeng He [1], Bang Shen [1], Rui Fang [1] *

[1] State Key Laboratory of Agricultural Microbiology, College of Veterinary Medicine, Huazhong Agricultural University, Wuhan, Hubei Province, 430070, People's Republic of China.

[2] College of Veterinary Medicine, Henan Agricultural University, Zhengzhou, Henan Province, 450046, People's Republic of China.

Correspondence: fangrui@mail.hzau.edu.cn

TableS1 Parasite strains used in this study.

Strain	Description	Source
TATi	Parental strain of i*Tg*AAC1	(1)
RH*Δku80*	Parental strain of location strain	From the Sibley Lab
i*Tg*AAC1	*Tg*AAC1 conditional knockdown	This work
Com-*Tg*AAC1	*Tg*AAC1 expressed in i*Tg*AAC1	This work
Com-*Mus*ANT2	*Mus*ANT2 expressed in i*Tg*AAC1	This work
RH*Δhxgprt*	Parental strain of RH*Δtgaac2*	From the Sibley Lab
Δtgaac2	*TgAAC2* deleted in RH*Δhxgprt*	This work

TableS3 Plasmids used in this study.

Plasmid name	Use	Source
pSAG1-Cas9-sg*UPRT*	Template for gene-specific CRISPR plasmid construction	(2)
pSAG1-Cas9-sg*TgAAC1*-lo	*TgAAC1* located-specific CRISPR plasmid	This work
pSAG1-Cas9-sg*TgAAC2*-lo	*TgAAC2* located-specific CRISPR plasmid	This work
pSAG1-Cas9-sg*TgAAC1*	*TgAAC1*-specific CRISPR plasmid	This work
pSAG1-Cas9-sg*TgAAC2*	*TgAAC2*-specific CRISPR plasmid	This work
p7TetOS1	Template for SAG1-TetO7 promoter amplification	From the Sibley Lab
pUC19	Template for pUC19 amplification	From the Sibley Lab
pTet-off :: *TgAAC1*-Ty	To construct the i*Tg*AAC1 strain	This work
p*TgAAC2* :: *CAT*	To replace *TgAAC2* with *CAT*	This work
pTub ::*Tg*AAC1::HA::*CAT*	To complement *Tg*AAC1 in i*Tg*AAC1	This work
pTub ::*Mus*ANT2::HA::*CAT*	To complement *Mus*ANT2 in i*Tg*AAC1	This work
pET-16b	Template for prokaryotic expression of *Tg*AACs	This work
pET-16b-*Tg*AAC1-HA	The prokaryotic expression plasmid of *Tg*AAC1	This work
pET-16b-*Tg*AAC2-HA	The prokaryotic expression plasmid of *Tg*AAC2	This work

1. Meissner M, Schlüter D, Soldati D. 2002. Role of Toxoplasma gondii myosin A in powering parasite gliding and host cell invasion. Science 298:837-40.
2. Shen B, Brown KM, Lee TD, Sibley LD. 2014. Efficient gene disruption in diverse strains of Toxoplasma gondii using CRISPR/CAS9. mBio 5:e01114-14.

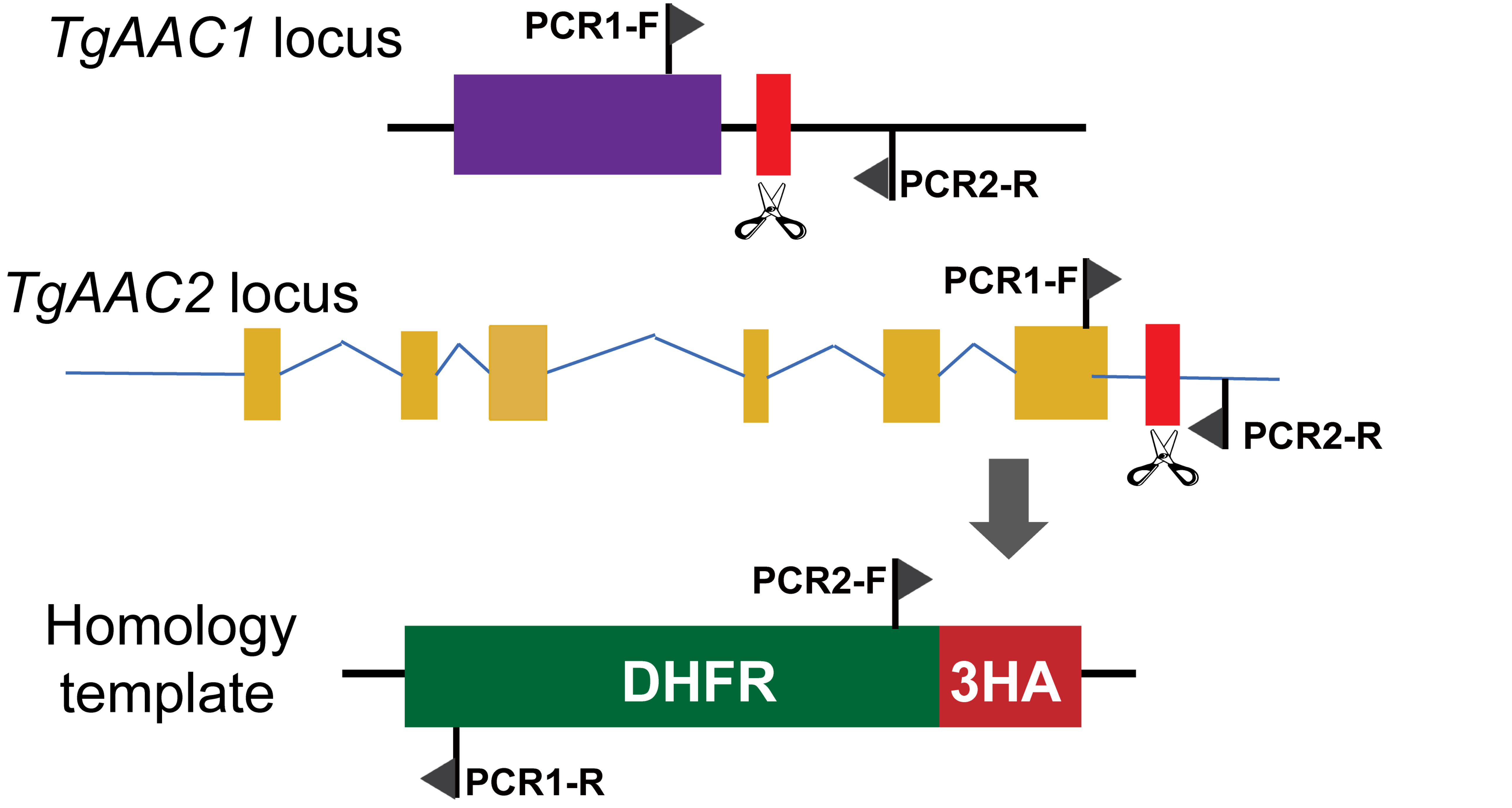

S-1. The strategy used to detect the localization of *Tg*AACs. DHFR were used as selection marker. *Tg*AAC1: PCR1-1174 bp, PCR2-1052bp. *Tg*AAC2: PCR1-809bp, PCR2-659bp.

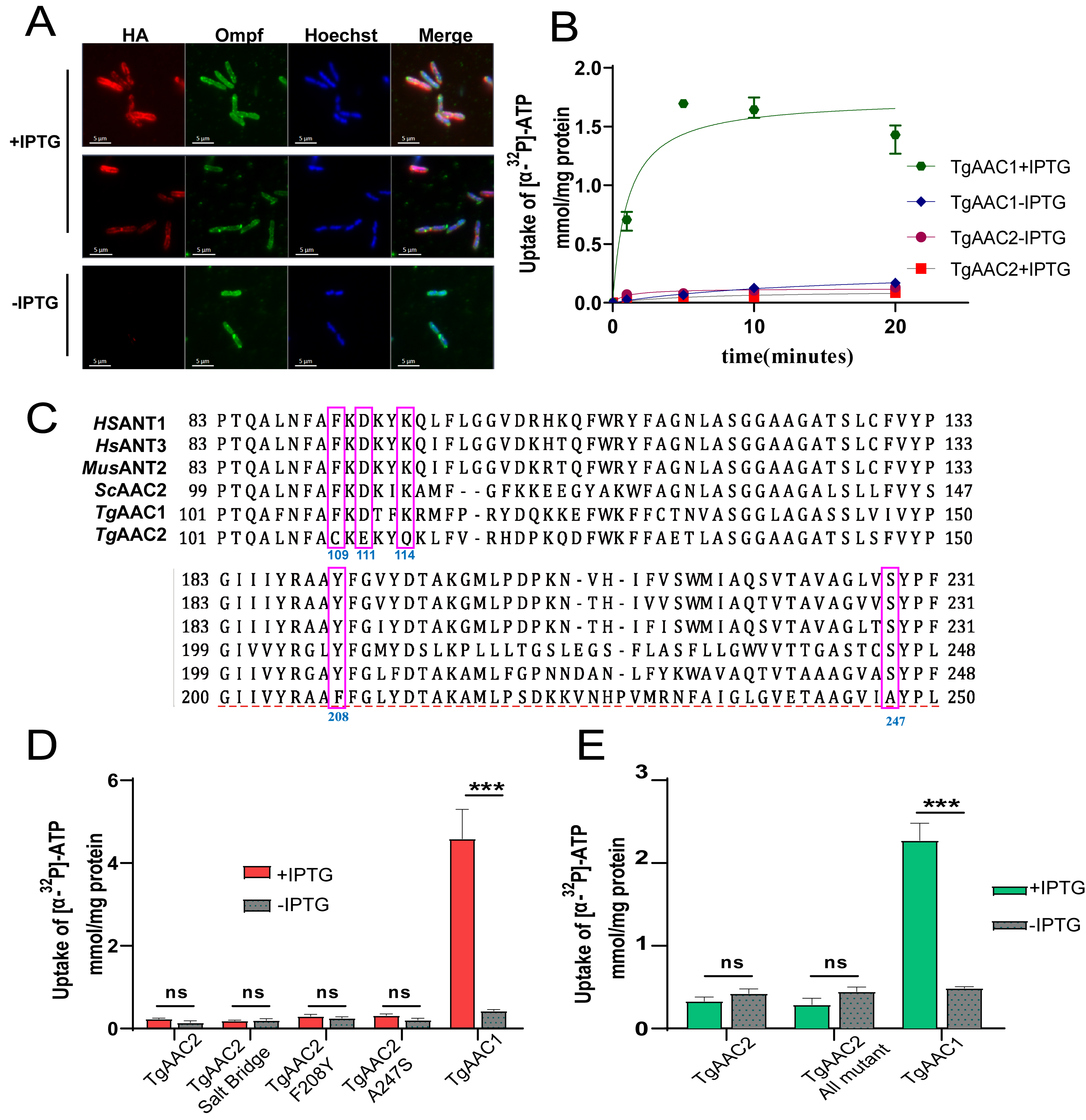

S-2. Functional identification of *Tg*AAC2 and the mutants. (A) Detection of *Tg*AAC2 expression and localization in *E. coli* by IFA using antibodies against HA tag, Ompf (outer membrane porin F) and Hoechst. (B) IPTG-induced *E. coli* cells harboring the plasmid encoding *Tg*ANT2 were incubated with [α-^{32}P]-ATP for the indicated time intervals. IPTG-induced or noninduced *E. coli* cells harboring the plasmid encoding *Tg*AAC1 were used as control. Means ± SD, data of three independent repeated. (C) The different amino acids of *Tg*AAC2 are circled in pink. (D and E) *E. coli* with mutations of *Tg*AAC2 were incubated with [α-^{32}P]-ATP for 10 min. *Tg*AAC2 Salt-bridge, with C109F, E111D, Q114K mutated. Means ± SD, data of three independent repeated.
